# CD11b promotes the differentiation of osteoclasts induced by RANKL through the spleen tyrosine kinase signalling pathway

**DOI:** 10.1111/jcmm.13254

**Published:** 2017-06-29

**Authors:** Guoxi Yang, Xiaoyong Chen, Zhao Yan, Qingsheng Zhu, Chongfei Yang

**Affiliations:** ^1^ Institute of Orthopaedic Surgery Xijing Hospital The Fourth Military Medical University Xi'an China

**Keywords:** bone micro‐CT, molecular pathways, cytokines, osteoclasts

## Abstract

Macrophage surface antigen‐1 (Mac‐1, CD11b/CD18) has been implicated in the regulation of osteoclastogenesis. In the synovial tissues of patients with aseptic loosening after total hip replacement, CD11b was up‐regulated, which indicated that CD11b is closely involved in osteolysis around the prosthesis. We found that CD11b, but not CD18, promoted osteoclast (OC) maturation. Here, we show CD11b up‐regulated the levels of spleen tyrosine kinase (Syk), c‐Fos and nuclear factor of activated T cells, cytoplasmic‐1 (NFATc1), as well as the activity of extracellular‐regulated kinase (Erk), and as a result, osteoclast precursors (OCPs) differentiated and became tartrate‐resistant acid phosphatase (TRAP)‐positive. In addition, increased tumour necrosis factor‐α (TNF‐α) induced by ultra‐high molecular weight polyethylene (UHMWPE) particles up‐regulated the level of CD11b. Taken together, these findings suggest that CD11b is a positive regulator of osteoclastogenesis and that it functions by activating the Syk signalling pathway, while CD18 does not have the same effect.

## Introduction

Total joint replacement surgery brings marked symptomatic relief with substantial functional improvement. However, prosthetic implants can also cause a variety of possible complications. While failure due to sepsis, fracture and dislocation has become relatively rare, particle‐induced osteolysis has been a major cause of aseptic loosening after total joint replacement. It is estimated that over 70% of implant failures are caused by aseptic loosening, and UHMWPE particles have been found to correlate with osteolytic lesions significantly 1. Aseptic loosening is initiated by an aseptic inflammatory response to the phagocytosis of implant wear particles, and OCs play the key role in this pathological process. Although Mac‐1 has been shown to be involved in the differentiation of OCs 2, the downstream mechanisms remain unexplored.

## Materials and methods

### Cell culture

OCPs were obtained from the spleens of 4‐week‐old C57BL/6J mice purchased from The Jackson Laboratory (Bar Harbor, ME, USA). Briefly, the spleens were cut into small pieces using surgical sterile scissors and compressed using the plunger of a 1‐ml syringe in circular motion against bottom of the petri dish, until no macroscopic pieces of the spleen were visible. The suspension of cells was filtrated through a 40‐μm nylon mesh (Fisher Scientific; 22363547, Nepean, ON, Canada) and transferred to a 50‐ml tube to which 8 ml of culture medium was added. The spleen cell suspension was centrifuged at 1000 r.p.m. for 5 min., after which the cell pellet was redissolved in 2 ml of red blood cell (RBC) lysis buffer (Sigma‐Aldrich, St. Louis, MO, USA; R7757). The suspension was then incubated for 10 min. on ice and washed two times by centrifugation at 1000 r.p.m. for 5 min. with 10 ml of medium. Flow cytometry was used for the identification of OCPs, and most of the cells were OCPs (B220^‐^CD3^‐^NK1.1^‐^CD11b^−/low^CD115^+^CD117^+^). The OCPs were added at a seeding density of 1 × 10^4^ cells per cm^2^ to 6‐well, 48‐well and 96‐well culture plates, and bone slices (Boneslices, Denmark) were added to a 96‐well culture plate in advance for bone resorption assays. The cultures were fed every 2 days with fresh α‐MEM medium containing M‐CSF (10 ng/ml, Sigma‐Aldrich) and soluble RANKL (sRANKL, 100 ng/ml; Sigma‐Aldrich).

### Antibody neutralization

Rat IgG2a and antibodies (anti‐CD11b, M1/70; anti‐CD18, M18/2; Abcam, Cambridge, England) were added to the medium at a concentration of 10 μg/ml after exchanging the medium 2.

### Lentivirus infection

Lentivirus (GeneCopoeia Inc., Rockville, USA) carrying *Itgam* shRNA (shCD11b‐1, shCD11b‐2) and empty lentivirus (Mock) were added to the medium (multiplicity of infection index, MOI = 10) after OCPs were seeded in the plate.

### TNF‐α stimulation

The medium was supplemented with TNF‐α (Sigma‐Aldrich) up to 100 ng/ml to stimulate OCPs for 15 min. before the addition of sRANKL 3.

### TRAP staining and analysis of bone resorption

TRAP‐positive cells were detected using a TRAP staining kit (Sigma‐Aldrich) according to the manufacturer's instructions. The number of TRAP‐positive cells (OCs) in 48‐well plate was determined at a magnification of 40× by a microscopy (Carl Zeiss, Jena, Germany), and then, the mean number of TRAP‐positive osteoclasts per well of each group was calculated 4. Bone slices were washed with PBS, fixed in 2.5% (vol/vol) glutaraldehyde and observed using a scanning electron microscope (EM) (JEOL JSM‐35CF, Tokyo, Japan). Bone resorption area of each slice was measured using a system consisting of a PC and image analysis software (Image J software, version 1.49n, NIH Image), and then, the relative bone resorption area per osteoclast of each group was calculated 5.

### RT‐PCR and quantitative real‐time PCR

Total RNA was extracted from the cells according to the manufacturer's protocol for TRIZOL reagent (Invitrogen, Carlsbad, CA, USA). Briefly, for total RNA extraction, cells cultured in 6‐well plate were mixed with liquid nitrogen and treated with TRIZOL reagent, at a proportion of 1 ml TRIzol for each well, following manufacturer's instructions. 500 ng of total RNA was used for reverse transcription (TaKaRa, Dalian, Japan), and, then 80 ng of cDNA samples was amplified with SYBR^®^ Premix Ex Taq™ II (TaKaRa). All data were analysed by the comparative Ct method (CFX96TM Real‐Time PCR Detection System; Bio‐Rad, Hercules, CA, USA) 2. CD11b‐specific primers were 5′‐GAGAACTGGTTCTGGCTTGC‐3′ (forward) and 5′‐ TCAGTTCGAGCCTTCTT ‐3′ (reverse); Syk‐specific primers were 5′‐AGGAAACCTCCACTTGCTCTC‐3′ (forward) and 5′‐GTCTGCACCCCTTCAGAGTT‐3′ (reverse); c‐Fos‐specific primers were 5′‐AGCCAGTCAAGAGCATCAGCAA‐3′ (forward) and 5′‐GCTCCCAGTCTGCTGCATAGAA ‐3′ (reverse); NFATc1‐specific primers were 5′‐CAACGCCCTGACCACCGATAG‐3′ (forward) and 5′‐GGCTGCCTTCCGTCTCATAGT‐3′ (reverse); and GAPDH‐specific primers were 5′‐TACAGCAACAGGGTGGTGGAC‐3′ (forward) and 5′‐GTGGGTGCAGCGAACTTTATT‐3′ (reverse). The mRNA levels of all genes were normalized to those of GAPDH in each sample (using the ΔΔCt method). The results are expressed as fold change relative to the control 6.

### Western blot analysis

Cells or periostea were lysed in 1X RIPA (Millipore, MA, USA). A BCA Protein Assay Reagent Kit (Pierce, MO, USA) was used to measure protein concentrations. Total protein was transferred to nitrocellulose membranes (Bio‐Rad) and incubated overnight with primary antibody: rabbit polyclonal anti‐Syk, anti‐P‐Syk (phospho‐Y352), anti‐c‐Fos, anti‐Erk1/2, anti‐P‐Erk, anti‐NFATc1, anti‐CD11b and anti‐β‐actin (Cell Signaling Technology, Boston, USA). Blots were then incubated with horseradish peroxidase (HRP)‐conjugated secondary antibody (donkey anti‐rabbit IgG, Jackson Immunoresearch, Baltimore, USA) in blocking buffer. Quantification of the immunoblots was performed using Image J software (version 1.49n, NIH Image) after exposed to X‐ray film, and results were normalized to β‐actin 7.

### Detection of serum TNF‐α

At 2 weeks after surgery, all of the mice were killed in a CO_2_ chamber. Blood was collected from the heart, and serum was collected after centrifugation. Serum TNF‐α was detected with corresponding ELISA kit (R&D Systems, Minneapolis, MN, USA) according to the manufacturer's instructions.

### UHMWPE particles

UHMWPE particles were provided by Mr. Ernst Krendlinger, the manufacturer of Clariant (Gersthofen, Germany). Ninety per cent of the particles were less than 9 μm in diameter, with diameters ranging from 0.08 to 11.13 μm (S.E.M. = 1.31 μm), with a mean size of 1.72 μm 8. The particles were resuspended in sterile phosphate‐buffered saline (PBS) at a concentration of 1 × 10^9^ particles/ml 9. The Limulus assay (Sigma‐Aldrich) was used to determine that the particle suspension was negative for endotoxin.

### Animal experiments and histologic analysis

Institutional approval was obtained for experiments about animals and specimens from human, and all experimental procedures involving animals and human specimens were in accordance with the institutional review board of The Fourth Military Medical University. Twenty‐four female 8‐week‐old C57BL/6J mice (wild‐type) and twelve female 8‐week‐old *Itgam‐*knockout (*Itgam*‐KO, B6.129S4‐Itgamtm1Myd/J) mice on a C57BL/6J background were purchased from The Jackson Laboratory. Twelve of the C57BL/6J mice received sham operation (sham control), and the intact periostea on the surface of calvaria were reserved. After implantation of 100 μl PBS on the calvarium, the incision was sutured. The rest of the twelve C57BL/6J mice (WT group) and *Itgam*‐KO mice (*Itgam*‐KO group) were treated with UHMWPE particles. Briefly, after anesthetization, a 1 × 1 cm^2^ area of calvarial bone was exposed overlying the calvaria, and the intact periosteum was carefully reserved. Mice from WT group and *Itgam*‐KO group received 100 μl of UHMWPE suspension. The incision was then sutured. Water and food were given *ad libitum*. 2 weeks after the operation, all animals were killed in a CO_2_ chamber 10. For the mice from WT group and *Itgam*‐KO group, periostea were cut‐off from the edge and carefully isolated from the calvaria using a tweezer; then, the periostea were used for Western blot. Mice calvaria from all of the three groups was used for histologic evaluation after a micro‐CT scan.

Similarly, twelve female 8‐week‐old C57BL/6J mice were randomly divided into two groups of six mice. The UHMWPE particles were implanted using the same procedures as described above: Briefly, in the sham control, the mice received 100 μl of PBS spread over the area, and the particles group received 100 μl UHMWPE particle suspension. After 2 weeks, all animals were killed in a CO_2_ chamber, periostea were harvested for Western blot assays and the blood was used to measure TNF‐α using a mouse TNF‐α commercially available enzyme‐linked immunosorbent assay (ELISA) kit.

Synovial tissues from patients with aseptic loosening after total hip replacement and fracture around the hip were made into formalin‐fixed sections (4 μm thick). Immunohistochemical staining was used to detect CD11b, and sections with over 50% of the cells stained brown were determined to be CD11b‐positive expression.

### Micro‐computed tomography analyses

High‐resolution *in vitro* micro‐computed tomography (Explore Locus SP; GE Healthcare, Madison, WI, USA) of the calvarial bone structure was performed on samples of mice in each group, and bone volume fraction (BVF) was used to measure the level of osteolysis 11.

### Histologic evaluation of osteolysis and osteoclast numbers

An Olympus BX‐51 microscope (Olympus, Tokyo, Japan) was used for osteolysis measurements. Sections were stained with haematoxylin and eosin (H&E) to determine the bone resorption area. In detail, at a magnification of 40x, bone resorption area of each section was measured by tracing the area of tissue including any bone resorption pits, and the area was recorded and calculated by the software (Image J software, version 1.49n, NIH Image). The mean resorption area of each section was calculated. TRAP staining was used for OC quantification. The image was oriented with the midline suture, which was 1.5 mm in diameter, in the centre of the field.

### Statistical analysis

spss 13.0 (Armonk, NY, USA) was used to perform statistical analysis. Significance was assessed by independent two‐tailed Student's t‐test or anova. All data in histograms of this study were presented as mean ± S.D. *P*‐values <0.05 were considered significant.

## Results

### CD11b blockage or deficiency decreased osteoclastogenesis

**Figure 1:**
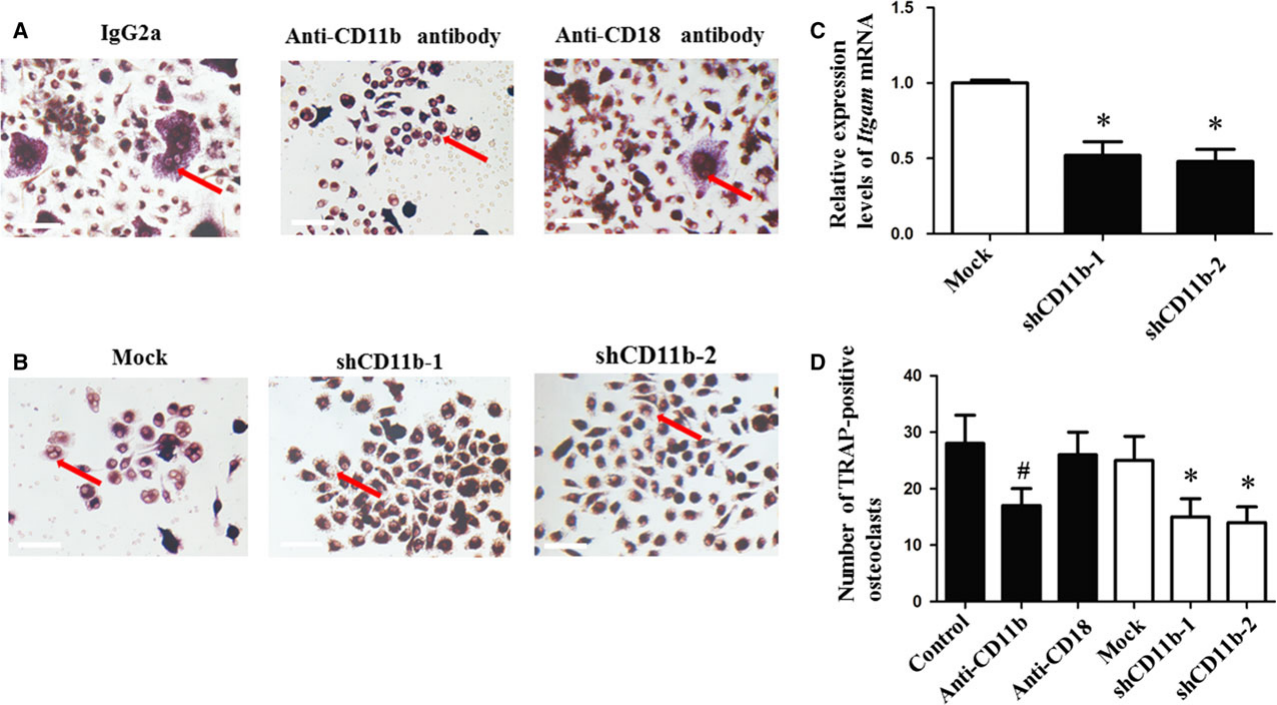
CD11b blockage or deficiency reduced the number of TRAP‐positive cells, while CD18 neutralization did not have the same effect. Mouse OCPs were treated with sRANKL (100 ng/ml) for 7 days. (**A** and **B**) TRAP staining. Red arrows refer to TRAP‐positive osteoclasts. (**C**) Relative expression levels of *Itgam* mRNA, *n* = 3. (**D**) Mean number of TRAP‐positive osteoclasts per well of different groups, *n* = 3. #*P* < 0.05 compared with the control group; *P < 0.05 compared with the mock group. Bar = 0.25 mm.

The number of TRAP‐positive cells neutralized by anti‐CD11b antibody was significantly lower than control and anti‐CD18 antibody group (Fig. 1A and D). *Itgam* mRNA levels were down‐regulated in lentivirus‐infected cells (Fig. 1C). The group infected with lentivirus contained a lower number of TRAP‐positive cells than mock group (Fig. 1B and D). However, no significant difference was observed between the groups treated with anti‐CD18 antibody and IgG2a (Fig. 1D).

**Figure 2:**
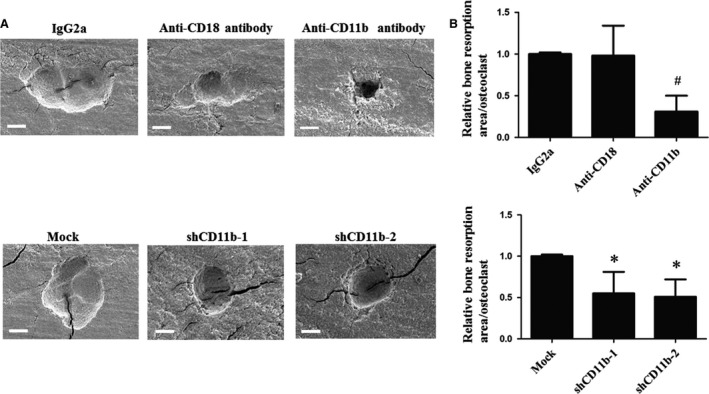
Scanning EM analysis of the effect of anti‐CD11b, anti‐CD18 antibodies and *Itgam* silencing on the bone resorption levels *in vitro*. Mouse OCPs were cultured on bone slices and treated for 7 days with sRANKL (100 ng/ml). (**A**) Bone resorption pits of cells treated with neutralizing antibodies (10 μg/ml) or lentivirus (MOI = 10). (**B**) Relative bone resorption area per osteoclast from different groups, *n* = 3. #*P* < 0.05 compared with the control group; *P < 0.05 compared with the mock group. Bar = 10 μm.

The effect of CD11b blockage or deficiency on bone resorption level was determined by scanning EM. Cells treated with anti‐CD11b antibody for 7 days presented lower resorption area per osteoclast than cells with the treatment of IgG2a and anti‐CD18 antibody. Similarly, cells infected with shCD11b lentivirus showed decreased bone resorption area per osteoclast than mock group (Fig. 2A and B).

### CD11b‐positive expression is correlated with aseptic loosening occurrence

**Figure 3:**
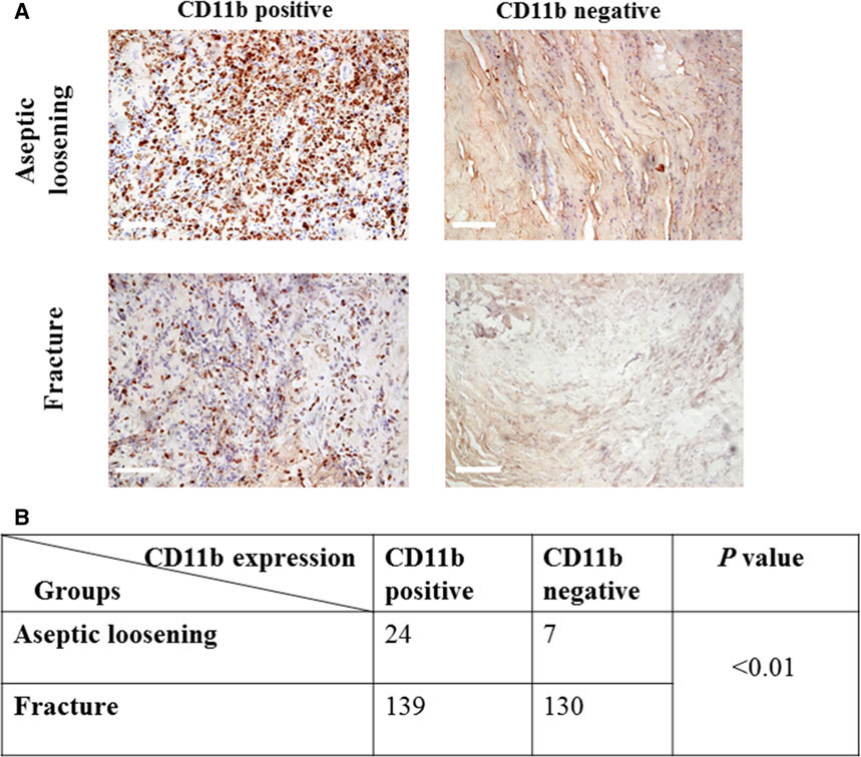
CD11b positive expression is correlated with aseptic loosening. (**A**) Immunohistochemical staining of synovial tissues from patients with aseptic loosening and fracture around the hip. (**B**) *Chi‐square* test result. Bar = 0.25 mm.

The expression of CD11b was examined using immunohistochemical staining. *Chi‐square* test result showed a highly significant correlation between the expression of CD11b and aseptic loosening (*P*  < 0.01). (Fig. 3).

### CD11b blockage or deficiency down‐regulated NFATc1 *via* the Syk signalling pathway

**Figure 4:**
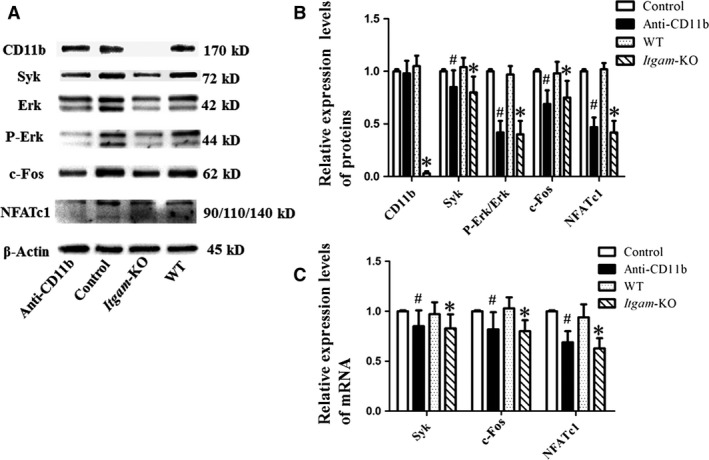
Western blot and real‐time quantitative PCR results showed that CD11b blockage or deficiency down‐regulated NFATc1 *via* the Syk signalling pathway. Mouse OCPs were treated with sRANKL (100 ng/ml) and M‐CSF (10 ng/ml), together with anti‐CD11b antibody (10 μg/ml) for 7 days. Periostea were from *Itgam*‐KO group and WT group. (**A**) Cell lysates and periosteum lysates were analysed by Western blotting. (**B**) Relative expression levels of proteins were presented as mean ± S.D., *n* = 3. (**C**) Total RNA was obtained for RT‐PCR and quantitative real‐time PCR, *n* = 3. #*P* < 0.05 compared with the control group; *P < 0.05 compared with the WT group.

According to Western blot results, levels of Syk, c‐Fos, NFATc1 and P‐Erk/Erk were lower in anti‐CD11b antibody treatment group than in the control group (Fig. 4A and B). Consistently, periostea from *Itgam*‐KO group exhibited little expression of CD11b and showed a down‐regulation of the proteins involved in the Syk signalling pathway (Fig. 4A and B). Similar results were found in the relative mRNA levels of Syk, c‐Fos and NFATc1 (Fig. 4C). The values of Ct ranged from 18 to 30.

### CD11b was up‐regulated by TNF‐α and UHMWPE particle burden promoted the expression of TNF‐α and CD11b

**Figure 5:**
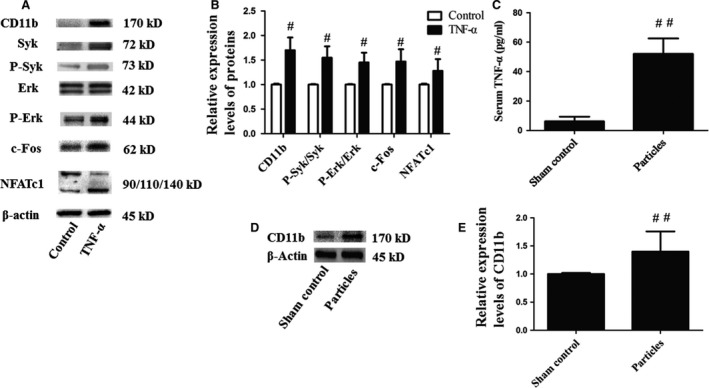
Western blot results and serum TNF‐α levels showed that CD11b was up‐regulated by TNF‐α and UHMWPE particle burden promoted the expression of TNF‐α and CD11b. Mouse OCPs were treated with sRANKL (100 ng/ml) and M‐CSF (10 ng/ml), together with TNF‐α (100 ng/ml). After 7 days of sRANKL treatment, Syk signalling pathway‐related proteins of cell lysates were analysed by Western blotting (**A** and **B**), *n* = 3. Serum TNF‐α levels in mice with or without UHMWPE particle burden were measured by ELISA kit (**C**), *n* = 6. Periosteum lysates were used for Western blotting to determine the level of CD11b (**D** and **E**), *n* = 3. #*P* < 0.05 compared with the control group; ##*P* < 0.05 compared with the sham control.

After stimulation with TNF‐α for 15 min., the level of CD11b in mouse OCPs was increased. Consistent with the results described above, the levels of CD11b, P‐Syk/Syk, c‐Fos, NFATc1 and P‐Erk/Erk were increased in the TNF‐α group (Fig. 5A and B). The serum TNF‐α of the particles group was nearly eightfold than that of the sham control group (Fig. 5C). Meanwhile, the level of CD11b in the periosteum was up‐regulated as shown in Western blot analysis (Fig. 5D and E).

### Knockout of *Itgam* inhibited the osteolysis induced by UHMWPE particles

**Figure 6:**
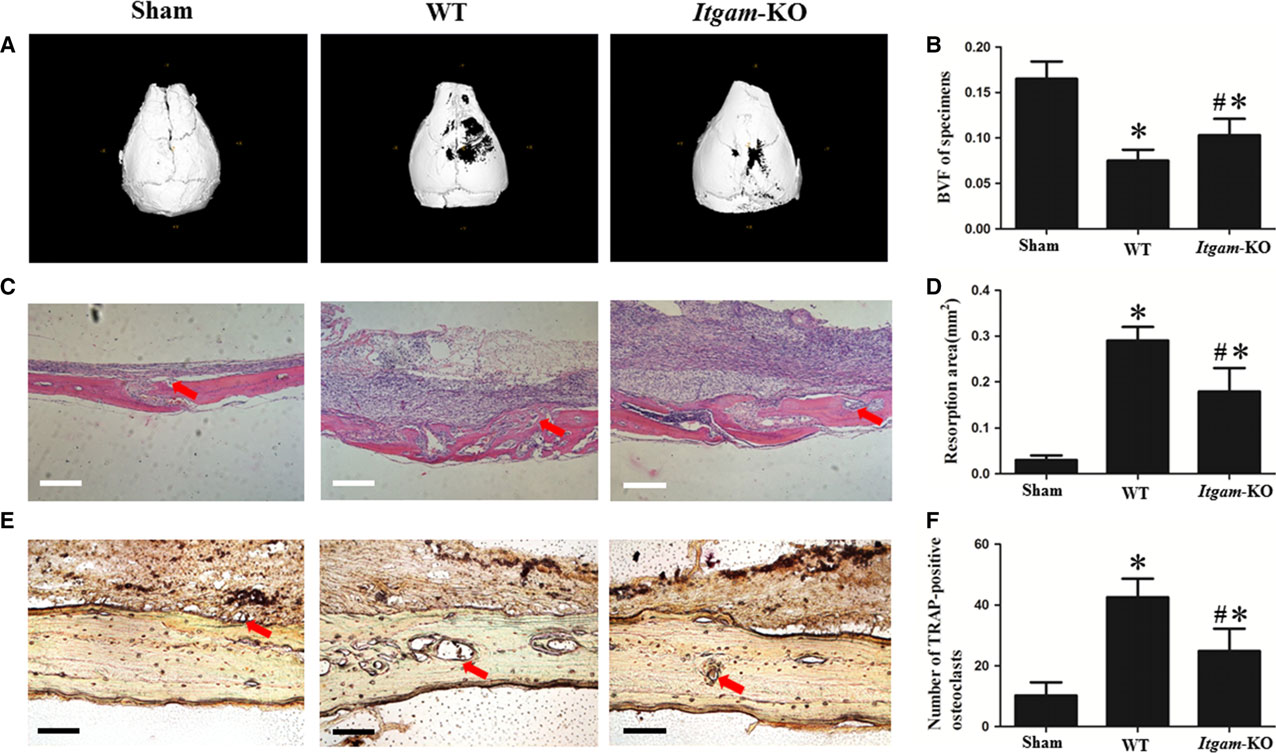
Micro‐CT three‐dimensional reconstruction images of the specimens and histologic staining of calvaria sections. (**A**) Digital reconstruction of specimens. (**B**) BVF of specimens. *n* = 12. (**C**) Haematoxylin and eosin staining of calvaria sections, red arrows refer to bone resorption areas, bar = 0.25 mm. (**D**) Average resorption area of each section, *n* = 12. (**E**) TRAP staining of calvaria sections, red arrows refer to TRAP‐positive OCs, bar = 50 μm. (**F**) Average number of TRAP‐positive OCs in each section, *n* = 12. ^#^ *P* < 0.05 compared with the WT group.*P < 0.05 compared with sham control.

Post‐operatively, all of the mice recovered from anaesthesia with no wound healing problems or other complications observed, and the animals were killed after 2 weeks. Although UHMWPE particle implantation resulted in osteolysis in the skulls of WT group and *Itgam‐*KO group in comparison with sham control, the inhibition of bone resorption in *Itgam‐*KO group could be easily observed (Fig. 6A and C). The TRAP‐positive OCs were stained purplish to dark red in the sections (Fig. 6E). One‐way anova analysis results showed that the bone volume fraction (BVF) of WT group was lower than *Itgam*‐KO and sham control (Fig. 6B). Additionally, bone resorption area and TRAP‐positive osteoclast number of WT group were higher than the other two groups (Fig. 6D and F).

## Discussion

Aseptic loosening after total joint replacement is the major underlying cause of joint revision, and bone resorption caused by osteoclasts plays an important role in the development of this pathological process 12.

This study indicates that CD11b promotes osteoclastogenesis. *Chi‐square* test result showed that specimens from patients with aseptic loosening exhibited up‐regulated CD11b, suggesting that CD11b significantly correlates with aseptic loosening. In the present study, *in vitro* results showed that anti‐CD11b antibody treatment had a negative impact on OC differentiation, while OCPs treated with anti‐CD18 antibody exhibited no significant reduction in OC formation rate. In our study of CD11b function in OCs using lentivirus, we found that *Itgam* silencing reduced the number of TRAP‐positive and multinucleate cells, which provides evidence for the role of CD11b in OC formation at the gene level. The loss of resorption function of OCs after treatment with anti‐CD11b antibody or lentivirus carrying *Itgam* shRNA likely indicates cells in a stage of undifferentiation. We also performed studies using UHMWPE particles, one of the most common and biologically relevant sources of wear debris in clinical practice 13, to more closely mimic the clinical scene. Based on the considerations above, in our *in vivo* experiments, bone resorption levels of WT group and *Itgam*‐KO group were significantly higher than sham control, indicating that the osteolysis models were successfully built. In addition, *Itgam*‐KO mice exhibited a marked inhibition of osteolysis and recruitment of osteoclasts under a burden of UHMWPE particles, which further reflected the profound involvement of CD11b in osteoclastogenesis.

Although the promotion of OC differentiation by CD11b was shown in our study, it is notable that we found no previous research showing the specific mechanisms underlying this effect. Our results demonstrate that CD11b acts as a positive regulator of OC differentiation through the downstream Syk signalling pathway. In the present study, neutralization of CD11b or knockout of *Itgam* led to down‐regulation of the levels of Syk, c‐Fos, NFATc1 and p‐Erk/Erk. Previous studies indicate that Syk is necessary for OC differentiation 14, 15, 16, and Erk1 plays an important role in OC formation, the phosphorylation of Erk1 results in increased bone resorption caused by osteoclastogenesis 17, 18, 19. Additionally, in the Jurkat T cell line and human‐purified T cells, the absence of Syk kinases correlated with defects in ERK phosphorylation and NFAT transcriptional activity 20. Besides, Myeung Su Lee *et al*. 19 found that c‐Fos expression is necessary for the expression of NFATc1. Taken together, these results suggest that CD11b promotes osteoclastogenesis through the up‐regulation of Syk, c‐Fos, NFATc1 levels and Erk activity.

In addition, we observed that TNF‐α stimulation up‐regulated the expression of CD11b in OCPs, and the levels of P‐Syk/Syk, P‐Erk/Erk, c‐Fos, NFATc1 in TNF‐α‐treated OCPs were increased. Besides, mice with up‐regulated TNF‐α showed increased CD11b expression. A previous study showed that TNF‐α induced an inflammatory response through a CD11b‐dependent mechanism. *In vitro* assessment of blood neutrophils showed a significant up‐regulation of CD11b after TNF‐α administration 21. Additional reports suggested that TNF‐α up‐regulates CD11b expression through diverse signalling pathways, including Src kinases, Akt, p38 MAPK and ERK1/2. Among the kinases under investigation, ERK 1/2 p44 is the pathway activated only by TNF‐α 22. All these findings demonstrate that the increased CD11b level in mice implanted with UHMWPE particles is caused by overproduction of TNF‐α and that TNF‐α may promote CD11b expression in OCPs *via* various signalling pathways. Hence, in consideration of this role for CD11b in osteolysis, TNF‐α is confirmed to exacerbate bone resorption through up‐regulation of CD11b.

In summary, our data suggest that CD11b promotes RANKL‐induced OC differentiation through the Syk signalling pathway. The high positive rate of CD11b expression in specimens from patients with aseptic loosening indicates the relevance of CD11b expression to particle‐induced osteolysis. Our experiments demonstrate that the neutralization of CD11b using a neutralizing antibody or the infection of a lentivirus carrying *Itgam* shRNA decreased the OC formation rate, while CD18 neutralization had no significant effect on OC differentiation. Besides, neutralization or deficiency of CD11b caused decreased bone resorption activity of OCs. In addition, knocking out *Itgam* inhibited osteolysis and OC recruitment in mice under a burden of UHMWPE wear particles. We further suggest that it is through the Syk pathway that CD11b promotes OC maturation. The deficiency of CD11b down‐regulated the expression of Syk, c‐Fos and NFATc1 and Erk phosphorylation. In our study, TNF‐α was shown to be a positive regulator of CD11b in OCPs and promoted osteolysis. Treatment with TNF‐α up‐regulated CD11b in OCPs, and mice with up‐regulated TNF‐α showed increased CD11b expression in periosteal tissue. The present findings suggest a regulatory role for CD11b in OCs that may provide a therapeutic target in osteolytic bone diseases.

## Conflict of interest

The authors report they have no conflicts of interest.
